# Psychosocial and Pharmacological Therapies to Reduce Alcohol Consumption in Severe Alcohol-Related Hepatitis Patients: A Case Report

**DOI:** 10.7759/cureus.37443

**Published:** 2023-04-11

**Authors:** Humza Awan, Nikhil Vergis

**Affiliations:** 1 Department of Metabolism, Digestion and Reproduction, Imperial College London, London, GBR

**Keywords:** alcohol-related hepatitis, cognitive behavioral therapy (cbt), alcohol dependence, alcohol addiction, addiction psychiatry, psychosocial therapy

## Abstract

Alcohol-related hepatitis (ARH) is an inflammatory liver disease caused by excessive alcohol intake over time. This represents a major health burden with a high mortality and poor prognosis. Reducing alcohol consumption is key to improving health outcomes and long-term mortality. Therefore, various measures have been implemented to aid in the reduction of alcohol consumption. On a population level, this includes minimum unit pricing to reduce alcohol purchases. On a patient level, evidence-based psychosocial and pharmacological therapies aid in achieving and maintaining alcohol abstinence, which will be explored through this case report.

A 39-year-old male with a four-year history of alcohol excess was admitted to a regional hospital. He presented with acute onset jaundice and examination findings were consistent with signs of chronic liver disease including abdominal distension and confusion. Investigations supported a diagnosis of severe ARH in this alcohol-dependent patient. Upon discharge, the patient received regular online cognitive behavioral therapy (CBT) sessions to aid in his abstinence.

Psychosocial therapy for alcohol abstinence can be categorized into brief and extended interventions. Brief interventions are short counseling sessions, which may be most effective in non-alcohol-dependent patients, whereas extended therapies including CBT, motivational enhancement therapy, and 12-step facilitation are longer regular therapies that may be more effective for alcohol-dependent patients. Some pharmacotherapies are contraindicated in ARH patients due to their hepatotoxicity and liver metabolism. However, acamprosate and baclofen are appropriate and effective treatments. Combining psychosocial and pharmacological therapy may be more beneficial than individual treatments to achieve and maintain abstinence.

## Introduction

Alcohol-related hepatitis (ARH) is an inflammatory liver disease caused by excessive alcohol intake. This represents a major cause of mortality [1]. Reducing the burden of ARH relies on reducing alcohol consumption [1]. Evidence-based treatments to aid in alcohol abstinence are explored in this case report.

## Case presentation

A 39-year-old male with a four-year history of alcohol excess (>14 units/week) was admitted to a regional hospital. The patient had no prior alcohol-related admissions. Full patient consent was acquired for this report.

On examination, he had signs of chronic liver disease including abdominal distension, scleral jaundice, ascites, asterixis, coagulopathy, and confusion. An abdominal CT scan revealed liver cirrhosis, portal hypertension, and moderate ascites. Initial treatment consisted of paracentesis, alcohol detox using benzodiazepines, and best supportive care including diuretics, antibiotics, and nutritional support.

He presented with acute onset painless jaundice lasting 10 days consistent with ARH rather than chronic progressive liver failure from cirrhosis. Hepatocellular carcinoma and cancer of the pancreatic head were excluded by CT scan and negative tumor marker serology.

On transfer to a liver unit six days after admission, blood tests showed: a raised white cell count, low creatinine, prolonged prothrombin time, raised aspartate aminotransferase (AST), alanine aminotransferase (ALT), and raised bilirubin (Table 1).

**Table 1:** Patient blood test results six days post-admission

Blood test	Results	Reference range
White cell count	12.6×10^9^/L	3.6-11.0 x 10^9^/L
Creatinine	58 μmol/L	59-104 μmol/L
Prothrombin time (PT)	19.8 seconds	10-14 seconds
Aspartate aminotransferase (AST)	120 IU/L	1-45 IU/l
Alanine aminotransferase (ALT)	40 IU/L	7-40 IU/L
Bilirubin	462 μmol/L	<21 μmol/L

The AST:ALT ratio of 3:1 was consistent with ARH. ALT was not suggestive of a viral or drug-induced hepatitis where markedly raised ALT would be seen (ALT>1,000IU/L). He had a neutrophil:lymphocyte ratio (NLR) > 8, a marker for poor response to steroid therapy.

Further investigations including a septic screen, hepatitis B and C serology and autoimmune serology were negative, excluding other causes of hepatitis. The patient was started on empirical antibiotics (Tazocin/Amikacin). He developed type 1 respiratory failure and a CT pulmonary angiogram revealed bilateral pleural effusions; this was managed by furosemide offloading. Following this, creatinine reached 340μmol/L over the course of five days. He became oligoanuric and imaging revealed an unobstructed urinary tract, consistent with hepatorenal syndrome or acute tubular necrosis. This was managed with human albumin solution and terlipressin.

The patient's Maddrey’s discriminant function was > 32, confirming a diagnosis of severe ARH. He was admitted to the intensive therapy unit (ITU) on day 13, where he underwent hemodiafiltration. A trans-jugular liver biopsy confirmed cirrhosis and fatty liver hepatitis with Mallory-Denk bodies. Alongside a raised hepatic-venous pressure gradient of 13mmH_2_O, findings were consistent with ARH. After 10 days, renal replacement therapy was stopped, and bilirubin plateaued at 600μmol/L.

The patient was moved back onto the ward and assessed for liver transplantation by an alcohol specialist nurse. He denied illicit drug use and had no criminal or legal issues. He expressed insight into his condition and the impact it had on his family. Therefore, he remained motivated to engage with all medical and alcohol support. He received support from his wife to help in his alcohol abstinence, having had no previous failed attempts. A UK Model for End-Stage Liver Disease (UKELD) score > 49 was calculated, meeting the criteria for transplantation. However, three months of alcohol abstinence was required to be eligible. A Sustained Alcohol Use Post Liver Transplantation (SALT) score of 4 was calculated, suggesting the risk of relapse post-transplantation was low.

Accounting for the patient’s poor prognosis and limited treatment options, compassionate use of fecal microbiota transplantation was done. He was then discharged and remained engaged with community alcohol services, receiving regular online CBT sessions. He achieved abstinence for 4.5 months before developing spontaneous bacterial peritonitis and sepsis leading to decompensated alcoholic liver disease. Despite ITU support, the patient unfortunately died five months after initial admission.

## Discussion

This case presents a severe ARH patient with alcohol dependence, achieving and maintaining alcohol abstinence is integral to improving prognosis. Effective interventions are necessary to achieve this. The societal burden has led to the introduction of public health measures such as minimum unit pricing (MUP) to modulate alcohol consumption. Recently, Anderson et al. showed MUP was associated with decreased alcohol purchases [1]. On a patient level, treatment encompasses psychosocial and pharmacological therapies.

Psychosocial therapy is the mainstay therapy in achieving alcohol abstinence for most patients. This could be in the form of brief interventions (BI) or extended therapies such as CBT, motivational enhancement therapy (MET), and twelve (12)-step facilitation (TSF).

BI usually consists of short counseling sessions lasting up to 15 minutes using diverse approaches to change harmful drinking behaviors, typically through patient-centered motivational techniques. A meta-analysis by Kaner et al. found moderate-quality evidence that BI leads to 20g/week less alcohol consumption compared to controls in primary care settings [2]. Also, extended therapies had no greater impact on alcohol consumption compared to BI [2]. A meta-analysis by Platt et al. found moderate-quality evidence that neither content nor setting of the BI significantly modulated effectiveness, although brief advice was the most effective content in reducing alcohol consumption [3]. Evidence suggests that BI is not most impactful for dependent drinkers, but rather in non-treatment seeking non-dependent patients [4]. Extended therapies have a greater role in dependent patients [4]. Gleeson et al. found that mean alcohol dependence severity was higher in severe ARH patients compared to heavy-drinking controls, with 26% having severe dependency [5]. Therefore, both BI and extended therapies have an important role in ARH patients.

CBT is typically a multi-session intervention targeting cognitive and affective risks of alcohol use, providing tools to reduce harmful behaviors and maintain abstinence. A meta-analysis by Magill et al. showed CBT to be significantly more effective than no or minimal treatment [6]. Due to the COVID-19 pandemic, the patient in this case received online-guided CBT sessions. Internet-delivered CBT has been shown to be non-inferior to face-to-face therapy in reducing alcohol consumption [7]. A randomized controlled trial (RCT) by Johansson et al. found that internet-based CBT significantly reduced weekly alcohol consumption (P=0.05) and that therapist-guided online CBT programs had no greater effect than self-help online CBT programs on alcohol abstinence [8]. Therefore, unguided internet-based CBT may be just as effective as traditional face-to-face-guided CBT treatments.

MET is patient-centered counseling concentrated on enhancing motivation to change harmful behaviors. An RCT by Sellemen et al. found MET effectively reduced unequivocal heavy drinking by 22% compared to minimal treatment arms (P=0.04) in patients with mild to moderate alcohol dependence [9]. Allen et al. found no significant difference between CBT and MET as individual treatments on three-year outcomes of alcohol abstinence. However, at both one year and three years' post-treatment, TSF conferred greater rates of total abstinence in comparison to CBT and MET [10].

TSF uses sets of guiding principles to outline actions to treat addiction and maintain abstinence. A meta-analysis by Kelly et al. found high-quality evidence that RCTs comparing professionally delivered TSF to CBT and MET found improved rates of continuous abstinence at 12 months (risk ratio: 1.21) with effects remaining consistent at 36 months [11]. This presents evidence that TSF may be more effective than CBT and MET for increasing abstinence. This meta-analysis also found moderate-quality evidence that for poor prognosis patients, TSF is more cost-effective than MET [11]. TSF may therefore improve both alcohol abstinence and cost-effectiveness in turn leading to greater treatment accessibility for patients.

A meta-analysis by Ray et al. found that combined CBT and pharmacotherapy were more effective than individual treatments alone with a small but significant effect size (g=0.28; P=0.03) observed in abstinence outcomes [12]. Also, comparing combined CBT-pharmacotherapy to combined MET-pharmacotherapy or TSF-pharmacotherapy showed no significant difference [12]. Therefore, combined therapy may enhance treatment effects. However, this meta-analysis included both alcohol and other substance use disorders.

Due to hepatic metabolism, many pharmacotherapies are contraindicated in ARH patients, drugs with low or no liver metabolism are hence most appropriate. Baclofen and acamprosate are two such drugs.

Baclofen is a gamma-aminobutyric acid (GABA) B-receptor agonist with low liver metabolism and no reported hepatic side effects. An RCT by James et al. found baclofen significantly reduced the number of cumulative heavy drinking days by 13.6 days (P=0.018) and increased abstinent days by 12.9 days (P=0.028) compared to placebo over 16 weeks [13]. Baclofen was also found to be more efficacious at higher doses (90mg/day) compared to lower doses (30mg/day), although higher doses also had lower tolerability [13].

Acamprosate modulates GABA and glutamate function in the brain and does not undergo metabolism therefore is appropriate for use in ARH patients. A meta-analysis of 24 RCTs by Rosner et al. found acamprosate compared to placebo significantly reduced the risk of alcohol consumption (risk ratio: 0.86) with a number needed to treat of 9.09, also that it increased abstinence duration by 11% [14].

An RCT found that CBT combined with acamprosate achieved lower rates of alcohol relapse (P<0.0005) and 24% greater abstinence at 12 weeks compared to CBT alone (P<0.006) [15]. Therefore, combined psychosocial and pharmacological therapy may lead to improved outcomes for alcohol abstinence, rather than either therapy alone.

## Conclusions

A variety of evidence-based treatments exist to aid in alcohol abstinence for ARH patients. Non-alcohol-dependent patients may find BI to be the most effective. However, dependent patients as highlighted in this case may require extended therapies or pharmacotherapy. There is compelling evidence for combining psychosocial therapy and pharmacotherapy in optimizing treatment effects to achieve and maintain alcohol abstinence.
